# Preparation, Interaction Mechanism and Application of Functional Ionic Liquid-Mediated Protein Imprinting Technique

**DOI:** 10.3390/polym18101171

**Published:** 2026-05-09

**Authors:** Nan Zhang, Jinrong Zhang, Kaishan Yu, Yang Qiao, Pengfei Cui, Chengzhao Yang, Minglun Li

**Affiliations:** 1 College of Chemistry and Chemical Engineering, Xi’an University of Science and Technology, Xi’an 710054, China; 2 State Key Laboratory of Polymer Science and Technology, Changchun Institute of Applied Chemistry, Chinese Academy of Sciences, Changchun 130022, China

**Keywords:** ionic liquid, protein imprinting polymers, molecular imprinting technique, protein recognition, interaction mechanism

## Abstract

Protein recognition underpins advances in drug discovery, immunoassays, clinical diagnostics and biosensing. As a biomimetic alternative to natural receptors, molecularly imprinted polymers (MIPs) have been developed to emulate antibody–antigen complementarity by generating binding cavities that mirror the size, shape and functionality of target macromolecules through template-directed polymerization and subsequent template removal. However, protein imprinting has historically been hampered by low imprinting efficiency and limited selectivity, rendering conventional protein-imprinted polymers (PIPs) inadequate for many contemporary biomedical applications. Functional ionic liquids (ILs)—a class of designer solvents and materials distinguished by tunable structures, exceptional physicochemical properties and favorable biocompatibility—have emerged as versatile additives to address the principal limitations of traditional PIPs, including poor selectivity, sluggish mass transfer and destabilization of protein conformation. Here, we provide a systematic review of the multifaceted roles that ILs play within protein-imprinting systems, delineating their employment as template-anchoring motifs, functional monomers, cross-linkers, porogens and structural stabilizers, and evaluating the consequent effects on polymer architecture and recognition performance. We further probe the multiplicity of non-covalent interactions between ILs and template proteins—highlighting the synergistic modulation afforded by electrostatic forces, hydrogen bonding, hydrophobic interactions and π-π stacking—and consider how such interplay can be harnessed to fine-tune binding-site fidelity. Consolidating recent progress, we summarize IL-enabled PIP applications in protein-specific recognition, biosensor development and analysis of complex real-world samples, and we critically examine the prevailing technical challenges and prospects for translation. The evidence indicates that ILs, by furnishing abundant interaction sites, accelerating mass transport and stabilizing native protein conformations, can markedly enhance PIP adsorption capacity, target specificity and recyclability, positioning them as a cornerstone for next-generation protein separation and enrichment materials and paving the way toward industrial deployment of protein-imprinting technologies.

## 1. Introduction

Proteomics is one of the leading disciplines in the field of life sciences, which has attracted considerable attention in biomedicine, immunoassay diagnosis, biosensing and other fields in recent years [1,2]. It is well known that in various practical applications related to proteins, such as protein-based vaccines and targeted drugs, the purity of proteins directly determines the performance of the final products [3]. Therefore, achieving efficient separation and purification of proteins is the top priority in the upstream protein industry [4,5]. To date, based on the inherent properties of proteins, including molecular size, isoelectric point and solubility, researchers have developed a variety of crude separation technologies for the initial separation of complex protein samples, such as membrane separation, precipitation and extraction methods. In addition, chromatographic separation, gel electrophoresis and chromatography technologies have also been successively developed and applied. However, the above-mentioned traditional separation and purification technologies have inherent defects, such as cumbersome operation procedures, high preparation costs and insufficient stability, which greatly limit their practical application [6,7]. Thus, there is an urgent need for new functional materials or novel technologies to promote the development of the protein separation and purification field.

As a novel bionic recognition technology, molecularly imprinted technology (MIT) constructs specific recognition sites in the polymer matrix with high precision, which are perfectly matched with the spatial structure and functional groups of template molecules through the template molecule-guided effect [3,8]. Endowed with prominent advantages including low preparation cost, excellent environmental stability, and reusability, MIT has exhibited broad application prospects in the fields of molecular separation and specific recognition [9,10]. Currently, the imprinting technology for small-molecule substances has been well-developed and mature [11]. However, when applied to proteins, characterized by large molecular weight, complex three-dimensional structure, and flexible conformation, traditional imprinting technology encounters obvious bottlenecks, such as low imprinting efficiency and poor specific recognition performance [12,13]. The key challenges mainly include three aspects: the large molecular size of proteins makes it difficult to form effective imprinting sites; the easy denaturation of proteins leads to the mismatch between imprinted cavities and target molecules; in addition, the poor aqueous compatibility of the imprinting system further restricts the practical application of protein imprinting technology [14]. In the field of protein molecular imprinting, template proteins can be immobilized in the imprinting system via covalent, semi-covalent, or non-covalent interactions [15]. Considering the subsequent template elution efficiency and the structural stability of imprinted polymers, the relatively weak non-covalent interaction has become the most widely used mechanism in the structural design of protein-imprinted polymers [16]. To overcome the aforementioned technical bottlenecks, researchers have developed a series of effective solutions, such as surface imprinting technology, antigenic determinant imprinting technology, and functional monomer modification [17,18]. These solutions have laid a solid foundation for the further development and application of protein-imprinted technology (PIT).

Ionic liquids (ILs) are defined as low-temperature molten salts that maintain a liquid state below 100 °C [19]. Typically composed of organic cations and organic/inorganic anions, ILs exhibit almost no vapor pressure under room temperature conditions [20]. This unique physical property, combined with their structural adjustability, distinguishes ILs from conventional functional materials and lays a foundation for their wide application in biomolecular separation. ILs possess prominent advantages, including adjustable cation-anion structures, strong biological activity, high stability, and excellent solubility [21]. These inherent merits enable ILs to be extensively applied in multiple fields, such as solvent extraction, chromatographic separation, organic catalysis, biosensing, and functional material synthesis [22,23]. In recent years, with the in-depth development of biomolecular recognition technology, ILs have gradually become a key material in protein separation and recognition research. In the field of biomacromolecule recognition and separation, numerous experimental studies have confirmed that ILs play a crucial role in regulating the conformational stability of biomacromolecules (e.g., proteins) [24]. Specifically, in the field of protein molecular imprinting, ILs can effectively stabilize the natural conformation of proteins, thereby addressing the core technical challenges of traditional protein imprinting [25,26]. According to the Hofmeister series, functionalized ILs can be rationally designed by introducing chaotropic cations and affinity anions [27]. These functionalized ILs can form multiple non-covalent interactions with proteins, including hydrogen bonding, hydrophobic interaction, and π-π stacking [28]. Such interactions enable ILs to wrap protein molecules effectively, protect the active sites of proteins, and significantly inhibit protein denaturation caused by external environmental factors, thus providing a stable microenvironment for the existence and function of biomacromolecules [29,30]. The integration of ILs into the protein molecular imprinting system provides an effective solution to the key bottlenecks of traditional protein imprinting technology [30]. These bottlenecks mainly include the easy conformational change in template proteins, low matching degree between imprinting sites and target molecules, severe non-specific adsorption, and insufficient adsorption capacity [12,31]. By introducing ILs, the adsorption capacity and specific recognition ability of imprinted materials for target proteins can be significantly improved, realizing precise imprinting and efficient separation of proteins [32]. This technical improvement not only provides a new approach for the design and preparation of high-performance protein separation materials but also lays a solid theoretical and technical foundation for the industrial application of protein molecular imprinting technology, promoting the practical application of protein separation and recognition technology in related fields.

Recently, ionic liquid-mediated protein imprinting technology has evolved into a core frontier in the field of biomacromolecule separation and recognition. The landmark reviews by Ding et al. and Liu et al. have laid an essential theoretical foundation for the development of this domain from the perspectives of biosensing and solid-phase extraction, respectively [28,33]. Compared with previously reported reviews, this work delivers three core incremental contributions: (i) six pivotal functional roles of ionic liquids in protein imprinting systems are systematically classified and elaborated, and the structure-activity relationship between ionic liquid structural design and imprinting performance is fully established; (ii) the synergistic regulation mechanism of multiple non-covalent interactions between ionic liquids and template proteins is deeply dissected, supplementing the microscopic mechanism governing the regulation of imprinting site fidelity; (iii) cutting-edge research advances in this field from 2020 to 2026 are comprehensively summarized, and the application scope is extended to a full-chain system covering bioseparation, biosensing, and clinical diagnosis. This review aims to provide systematic theoretical guidance and research insights for the rational design of high-performance ionic liquid-mediated protein imprinting materials.

Since 2020, the field has achieved systematic breakthroughs in four key directions. Firstly, theoretical computation and data-driven approaches have evolved from auxiliary mechanistic interpretation tools into front-end design strategies for imprinting systems: molecular dynamics simulations, machine learning, and molecular docking are now used to unravel the interaction mechanisms between ionic liquids and proteins and to guide the rational screening of functional monomers. Second, the molecular design of functionalized ionic liquids has expanded beyond conventional imidazolium-based skeletons to include tailored systems such as boronic acid-functionalized, amide-rich, and stimuli-responsive ionic liquids, enabling precise control over protein conformation and the fidelity of imprinting sites. Third, the deep integration of ionic liquids with advanced porous materials and smart responsive strategies has significantly improved the mass transfer efficiency, antifouling performance, and smart controllability of imprinting materials. Fourth, the application scenarios have extended from laboratory-scale bioseparation and purification to clinical diagnostics and targeted drug delivery, robustly promoting the translation of this technology.

**Figure 1:**
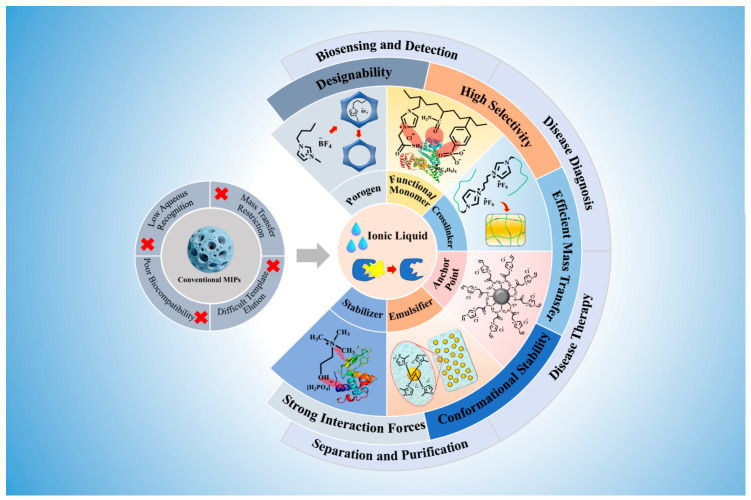
Schematic diagram illustrating the multifunctional roles, performance advantages, and application domains of ionic liquid-mediated protein molecularly imprinted polymers. The red crosses explicitly represent the key inherent limitations of conventional protein molecularly imprinted polymers, including insufficient aqueous-phase recognition, sluggish mass transfer, challenging template elution, and inferior biocompatibility.

Based on this, this review systematically summarizes the multifunctional roles of ILs in protein imprinting systems, detailing their specific applications and effects as template anchoring sites, functional monomers, cross-linkers, porogens, and structural stabilizers. It further explores the multiple non-covalent interaction mechanisms between ILs and template proteins, with an emphasis on the synergistic effects of electrostatic interactions, hydrogen bonding, hydrophobic interactions, and π-π stacking. In addition, it comprehensively reviews the latest advances in IL-mediated PIPs for protein-specific recognition, biosensor construction, and the analysis of complex real samples, while systematically analyzing current technical challenges and future development trends in this field (Figure 1).

## 2. Recent Advances in Ionic Liquid-Mediated Protein Imprinting

In recent years, research on ionic liquids in protein imprinting has exhibited a clear paradigm shift: ionic liquids are evolving from passive functional additives into programmable recognition units. This shift is particularly evident in four methodological dimensions: mechanistic understanding driven by theoretical computations, the incorporation of smart responsive properties, synergistic integration with nanomaterials, and the emergence of deep eutectic solvents (DES) as green alternatives.

### 2.1. Theoretical Computation-Driven Mechanisms of Ionic Liquid-Protein Interactions

The rational design of ionic liquid monomers and their interactions with proteins has long relied on empirical trial-and-error approaches, which are inefficient and lack transferability across different protein systems. The integration of theoretical computations and data-driven methods is now reshaping this landscape. Liu et al. developed a machine learning framework that combines molecular docking, unsupervised learning, molecular dynamics simulations, and correlation analysis to systematically unravel the microscopic mechanisms by which ionic liquids enhance membrane protein stability [34]. Their simulations reveal that ionic liquids form clusters on the protein surface and penetrate the hydration layer, effectively enhancing the structural stability of membrane proteins through the formation of an intermolecular hydrogen-bonding network. Furthermore, using supervised learning, the authors constructed a predictive model for protein stability; interpretability analysis validated the mechanistic findings and quantitatively elucidated the contributions of hydrogen bonding and interfacial architecture to membrane protein stability. This work provides a data-driven design framework for the rational screening of functional monomers in ionic liquid-based protein imprinting systems, highlighting the central value of theoretical computations in the upfront design of imprinting systems.

### 2.2. Thermoresponsive Ionic Liquids for Smart Responsive Imprinting Materials

**Figure 2:**
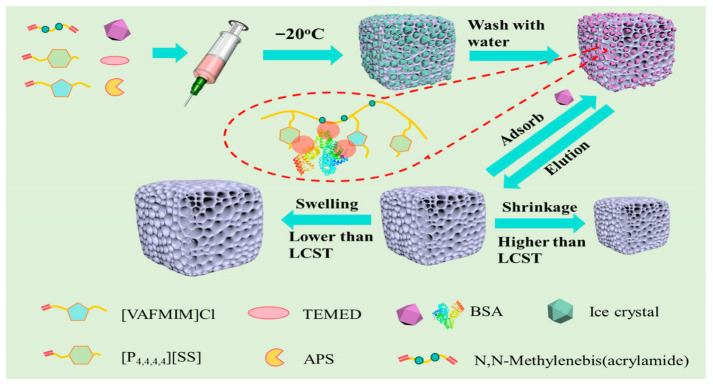
Preparation route of MICs. Reproduced with permission from [35], ACS Omega, American Chemical Society, 2025.

A major obstacle to the clinical translation of protein-imprinted materials lies in the harsh template elution conditions (e.g., sodium dodecyl sulfate or extreme pH buffers), which readily induce template protein denaturation and restrict their applications in drug delivery and continuous separation. Incorporation of thermoresponsive moieties into ionic liquid structures enables efficient protein capture under physiological temperature and mild elution via simple temperature modulation. As illustrated in Figure 2 [35], Cheng et al. utilized a lower critical solution temperature (LCST)-type IL as a thermosensitive functional monomer to fabricate bovine serum albumin (BSA)-specific molecularly imprinted cryogels through cryopolymerization. The subzero polymerization endowed the material with an interpenetrating macroporous network, delivering a high adsorption capacity of 745.1 mg/g, an adsorption equilibrium within 125 min, and an imprinting factor of 1.65. The cryogels displayed optimal adsorption performance at 37 °C, manifesting distinct thermoresponsive behavior. Moreover, the material retained 87% of its initial adsorption capacity after five adsorption–desorption cycles, exhibiting favorable reusability. This strategy integrates the structural tunability of ILs with smart responsive characteristics, offering a promising platform for the development of next-generation protein-imprinted materials toward on-demand recognition and controlled release.

### 2.3. Ultrasensitive Biomarker Detection Using Ionic Liquid-Nanomaterial Interfaces

**Figure 3:**
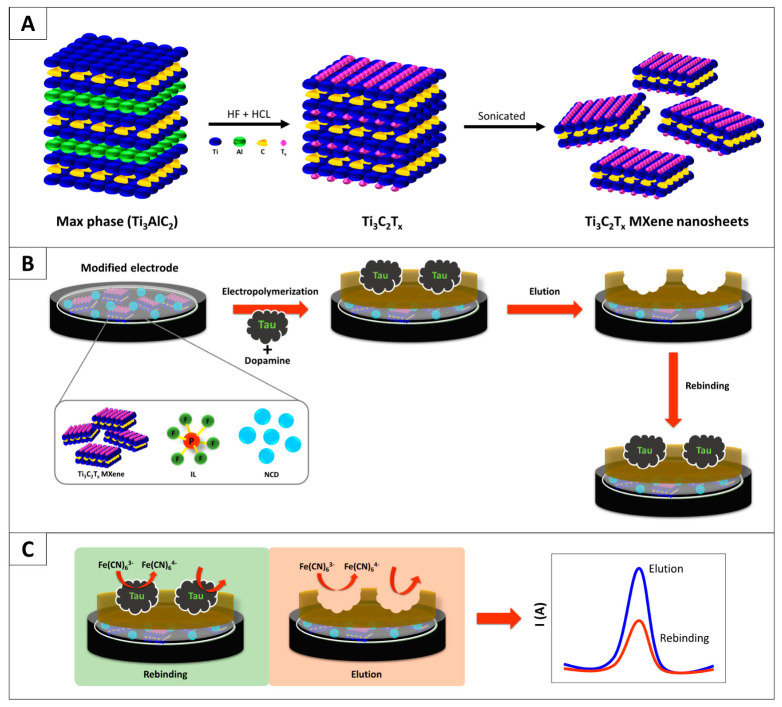
Schematic illustration of (**A**) Ti_3_C_2_T_x_ MXene nanosheet, (**B**) MIP/MXene-NCD/IL preparation, and (**C**) MIP detection process. Reproduced with permission from [36], Elsevier, 2025.

Precise detection of protein biomarkers associated with neurodegenerative diseases is critical for early clinical diagnosis. Ionic liquids serve a triple role in biosensing interfaces: modulating the electrode microenvironment, facilitating electron transfer, and stabilizing the three-dimensional architecture of the recognition layer. In recent years, the synergistic integration of ILs with advanced nanomaterials has substantially reduced the detection limits of sensing platforms. As illustrated in Figure 3 [36], Pakapongpan et al. fabricated a screen-printed electrochemical sensor co-modified with Ti_3_C_2_T_X_ MXene nanosheets, nitrogen-doped carbon dots and ILs for the selective detection of Tau protein, a pivotal biomarker of Alzheimer’s disease. In this sensing system, MXene delivers a large specific surface area and superior conductivity, whereas nitrogen-doped carbon dots and ILs improve the functionalization of the electrode interface and accelerate charge transfer within the polymer matrix. A polydopamine-based molecularly imprinted recognition layer exerts a synergistic effect with the nanocomposite to achieve high-specificity recognition. The sensor presents a linear response in the range of 10–300 pg/mL with an ultralow detection limit of 1 pg/mL, and its reliability and practical feasibility have been verified via spiked artificial serum tests. This design strategy can be extended to the detection of other disease-related biomarkers, revealing excellent versatility.

### 2.4. Deep Eutectic Solvents in Protein Imprinting

As a rapidly expanding subclass of ionic liquid analogues, deep eutectic solvents (DESs) have garnered substantial attention in protein imprinting, owing to their intrinsic merits of low toxicity, biodegradability, versatile physicochemical tunability and facile purification-free synthesis. DESs act as multifunctional components in imprinting systems, including functional monomers, comonomers, cross-linkers, porogens and green reaction media, providing a more sustainable and cost-effective alternative to conventional ionic liquids. Meanwhile, DESs can establish intensive non-covalent interactions with template proteins through hydrogen-bonding networks between hydrogen bond donors and acceptors.

**Figure 4:**
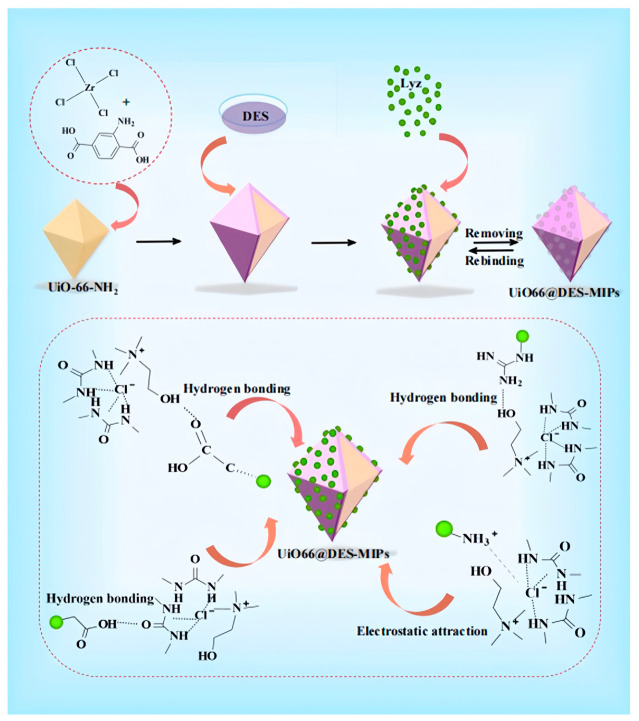
Synthesis of UiO66@ DES-MIPs and study of their mechanism of action. Reproduced with permission from [37], Springer, 2024.

As schematically illustrated in Figure 4 [37], Zhao et al. developed a water-compatible DES functional monomer consisting of choline chloride and 1,3-dimethylurea, and prepared lysozyme-imprinted polymers on UiO-66-NH_2_ metal–organic framework supports. The resultant material exhibited a remarkable adsorption capacity of 244 mg·g^−1^ toward lysozyme, with an imprinting factor of 3.67, and enabled efficient purification of lysozyme from real egg white samples. This work exemplifies the rational integration of DESs with porous supports for high-performance target protein recognition under mild aqueous conditions.

Moreover, Katrak and Ijardar systematically reviewed the applications of diverse DES-based systems for protein extraction and purification from plant, animal and food sources, highlighting their superiority over traditional organic solvents in extraction efficiency and preservation of protein native conformation [38]. Collectively, these studies confirm that DESs not only serve as green alternatives to ionic liquids but also represent a versatile and tunable platform for constructing high-performance next-generation protein-imprinted materials.

## 3. Multifunctional Roles of Ionic Liquids in Protein Imprinting

Owing to the high structural tailorability of anions and cations, ionic liquids can perform multiple core functions in protein imprinting systems. To clearly clarify the overall functional framework and prominently highlight their application value, this paper systematically summarizes the six core functional roles of ionic liquids in Table 1 in terms of functional classification, core functions and key advantages, which enables the precise regulation of template immobilization, polymerization microenvironment, pore structure and recognition performance [28].

### 3.1. Ionic Liquids as Protein Anchoring Sites

The immobilization efficiency and stability of template proteins in the pre-polymerization system directly determine the quantity, uniformity and recognition precision of imprinted sites [16]. Traditional physical adsorption and covalent bonding strategies are plagued by inherent limitations, including protein shedding, low loading capacity, heterogeneous site distribution, and conformational distortion of proteins [39]. As high-efficiency anchoring mediators, ionic liquids can firmly immobilize template proteins through multiple non-covalent interactions, restrain the random diffusion of templates, and substantially enhance the stability of pre-polymerized complexes [33].

**Figure 5:**
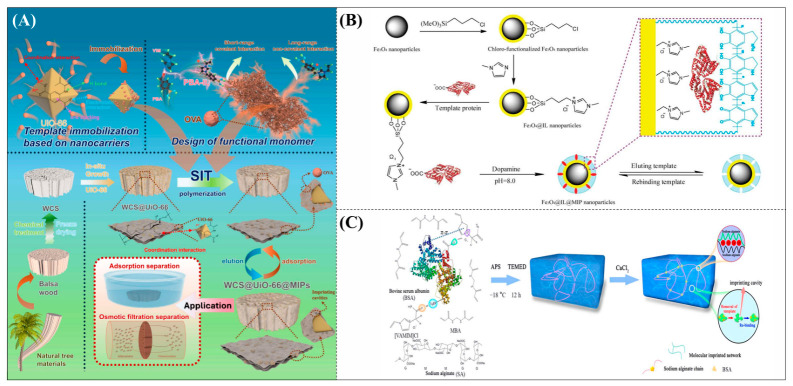
(**A**) Schematic diagram illustrating the principle, preparation process, and application mechanism of WCS@UiO-66@MIPs. (**B**) Schematic diagram of the synthesis of Fe_3_O_4_@IL@MIP nanoparticles. (**C**) Schematic diagram of the preparation process of MICM. Reproduced with permission from [40,41,42], Elsevier, 2025; Elsevier, 2017; MDPI, 2022.

Qian’s group constructed a glycoprotein imprinting system using a phenylboronic acid-functionalized imidazolium ionic liquid (PBA-IL) and a wood-derived cellulose sponge@UiO-66 composite support. PBA-IL was employed as an anchoring agent to enhance the immobilization efficiency of ovalbumin (OVA), as shown in Figure 5A [40]. This system established a dual-mode anchoring mechanism of “long-range capture and short-range recognition” through hydrogen bonding, electrostatic interactions, and π-π stacking between the imidazolium cations and OVA, synergized with reversible covalent bonds formed between the phenylboronic acid groups and the vicinal diol structures of the glycoprotein. Molecular simulation results demonstrated that the binding strength of PBA-IL to OVA was significantly superior to that of the conventional functional monomer 4-vinylphenylboronic acid. The resulting imprinted material, WCS@UiO-66@MIPs, exhibited an adsorption capacity of 571 mg/g for ovalbumin, an imprinting factor of 5.09, and an adsorption equilibrium time of only 40 min [40]. Hu’s group at Northwestern Polytechnical University prepared ionic liquid-functionalized Fe_3_O_4_@IL carriers by modifying magnetic nanoparticles (Fe_3_O_4_) with 1-methylimidazole, as shown in Figure 5B [41]. Density functional theory (DFT) calculations revealed that the carrier immobilized bovine serum albumin (BSA) through hydrogen bonding and π-π stacking interactions, achieving a protein loading capacity nearly nine times higher than that of bare Fe_3_O_4_ nanoparticles. Subsequently, an imprinted shell was fabricated via dopamine self-polymerization; an optimal shell thickness of 20 nm afforded the best selectivity and separation performance for the target protein [41]. Fan et al. constructed a poly (ionic liquid)-calcium alginate composite imprinted membrane (MICM) using the amide-functionalized ionic liquid 1-vinyl-3-carbamoylimidazolium chloride ([VAMIM]Cl) as both a protein anchoring site and a functional monomer, as shown in Figure 5C [42]. Taking advantage of the ion pairs, hydrogen bonds, and π-π stacking interactions provided by the imidazolium ring and amide groups, this ionic liquid anchored BSA within the polymer network, achieving stable immobilization and homogenization of template protein binding sites in an aqueous system. This ionic liquid markedly overcame the bottlenecks of conventional imprinting systems, including protein detachment, restricted mass transfer, and low recognition efficiency. The resulting imprinted membrane exhibited a maximum adsorption capacity of 485.87 mg/g for BSA, an imprinting factor of 2.16, and excellent separation selectivity in calf serum [42].

Therefore, the high designability of ionic liquids enables the optimization of interactions with template proteins through functional group regulation, achieving efficient protein anchoring. This strategy enhances protein loading capacity and the uniformity of imprinted sites by virtue of multiple non-covalent interactions. Meanwhile, the intrinsic properties of ionic liquids reduce non-specific adsorption, endowing imprinted materials with simultaneous improvements in adsorption capacity, selectivity, mass transfer rate and separation performance in practical biological matrices [33,43]. This provides a feasible solution to break through the bottlenecks of traditional protein imprinting.

### 3.2. Ionic Liquids as Functional Monomers

Functional monomers act as the core component governing the binding strength and recognition specificity of imprinted sites [8]. Conventional functional monomers merely rely on hydrogen bonding interactions and suffer from inherent drawbacks including a single interaction pattern, weak intermolecular interaction and poor aqueous compatibility [44]. By virtue of structural design, ionic liquid-based functional monomers can introduce diverse active groups, form multiple synergistic non-covalent interactions with amino acid residues on the protein surface, and markedly improve binding stability and recognition specificity [45].

**Figure 6:**
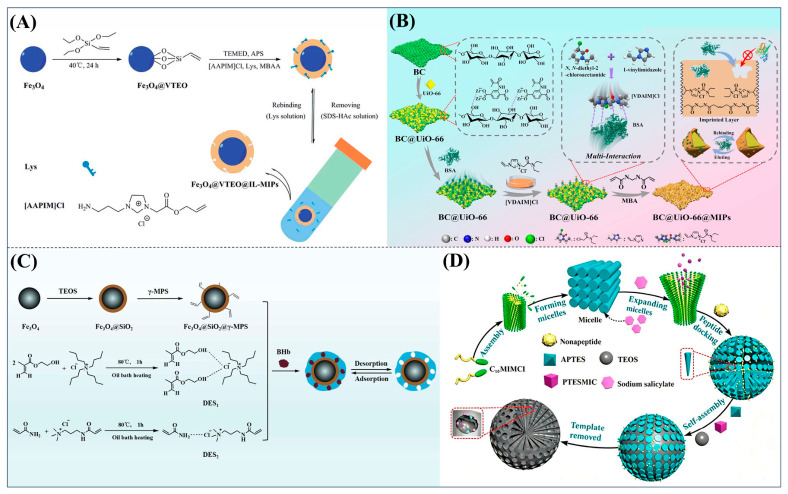
(**A**) Schematic diagram of the synthesis of MICs. (**B**) Schematic diagram of the synthesis of BC@UiO-66@MIPs. (**C**) Schematic diagram of the synthesis of DESs-MMIP. (**D**) Preparation procedure of epitope imprinted mesoporous silica. Reproduced with permission from [45,46,47,48], Elsevier, 2026; Royal Society of Chemistry, 2018; Elsevier, 2020; American Chemical Society, 2023.

For example, Xu et al. employed the imidazolium ionic liquid 1-(α-allylacetate)-3-(3-aminopropyl)imidazolium chloride ([AAPIM]Cl) as a functional monomer. Using surface imprinting technology on vinyl-functionalized Fe_3_O_4_ magnetic microspheres, they constructed core–shell magnetic molecularly imprinted polymers (Fe_3_O_4_@VTEO@IL-MIPs) for the selective separation of lysozyme (Lys), as shown in Figure 6A [45]. This ionic liquid monomer formed multiple binding sites with lysozyme through electrostatic interactions, hydrogen bonding, and π-π stacking involving the imidazolium cation, markedly enhancing the stability of the pre-polymerization complex. Under optimized conditions (200 mg of functional monomer, pH 7.1), the resulting imprinted material achieved a maximum adsorption capacity of 213.7 mg/g for Lys, an imprinting factor of 2.02, and an adsorption equilibrium time of only 2.5 h. Selective adsorption experiments demonstrated that the material recognized Lys significantly better than competing proteins such as bovine hemoglobin (BHb), BSA, cytochrome c (Cyt c), and OVA. Moreover, it successfully enabled the selective separation of Lys from real egg white samples, showing good practical application potential [45]. Qian’s group designed and synthesized the imidazolium-based ionic liquid 1-vinyl-3-diacetamidoimidazolium chloride ([VDAIM]Cl) as a functional monomer. Using a bacterial cellulose/UiO-66 hybrid scaffold as the support and combining it with surface imprinting technology, they prepared a high-affinity imprinted membrane (BC@UiO-66@MIPs), as shown in Figure 6B [46]. Spectroscopic characterization and molecular docking confirmed that [VDAIM]Cl formed multiple strong interactions with BSA, significantly enhancing the stability of the pre-polymerization complex. The resulting imprinted membrane exhibited a binding capacity of 502.5 ± 27 mg/g, an imprinting factor as high as 6.27 ± 0.84, a competitive adsorption factor of 5.57 ± 0.62 in a BSA/hemoglobin (Hb) mixture, and a permeation selectivity coefficient of 6.37, demonstrating excellent selective recognition ability [46]. Furthermore, the same team designed a boronic acid-containing imidazolium ionic liquid monomer capable of forming both reversible covalent bonds and multiple non-covalent interactions, further improving glycoprotein imprinting efficiency. Liu et al. used deep eutectic solvents as both a functional monomer and a cross-linker—namely, hydroxyethyl methacrylate/tetrabutylammonium chloride (DES_1_) and acrylamide/(3-acrylamidopropyl)trimethylammonium chloride (DES_2_)—to prepare magnetic molecularly imprinted polymers (DESs-MMIP) on the surface of silica-coated magnetic nanoparticles (Fe_3_O_4_@SiO_2_) via surface imprinting technology for the specific recognition of BHb, as shown in Figure 6C [47]. The DESs-MMIP exhibited a maximum adsorption capacity of 229.54 mg/g for BHb and an exceptionally high imprinting factor of 21.89. Its recognition ability for BHb was far superior to that for interferents such as BSA, OVA, pepsin (Pep), trypsin (Try), DNA, and amino acids. Moreover, the material successfully achieved selective separation of BHb from real calf serum, significantly outperforming imprinted materials constructed with conventional functional monomers and cross-linkers [47]. In addition, Cheng et al. developed the amphiphilic imidazolium ionic liquid 1-hexadecyl-3-methylimidazolium chloride (C_16_MIMCl) as a dual-functional monomer, serving both as a recognition unit and a micelle template for epitope imprinting. Using the C-terminal nonapeptide of Cyt c as the pseudotemplate and ordered mesoporous silica as the support, they prepared epitope-imprinted mesoporous silica (EIMS) via a mild sol–gel route, as shown in Figure 6D [48]. The imidazolium cation of C_16_MIMCl formed multiple non-covalent interactions with the peptide template, ensuring oriented assembly and high-fidelity recognition cavities, while sodium salicylate was introduced to tailor the mesopore size for efficient mass transfer. The resulting EIMS exhibited a maximum adsorption capacity of 249.6 mg/g for Cyt c, an imprinting factor of 3.8, and achieved rapid adsorption equilibrium within 10 min. It showed superior selectivity toward Cyt c over competing proteins and successfully separated Cyt c from fetal bovine serum samples, demonstrating good practical application potential [48].

In summary, ionic liquid-based functional monomers overcome the limitations of conventional monomers via structural design and multi-site interactions, improve the stability of pre-polymerized complexes and the precision of imprinted sites, and can further endow imprinted materials with stimuli-responsive properties. This provides a reliable strategy for the rational design of high-performance smart protein-imprinted materials [21].

### 3.3. Ionic Liquids as Cross-Linkers

Cross-linking agents exert a core function in immobilizing functional monomers and maintaining the three-dimensional structure of imprinted cavities [49]. Conventional cross-linking agents construct polymer networks merely through single covalent bonds, accompanied by drawbacks such as inhomogeneous cross-linking density and weak affinity for template proteins [50]. Ionic liquid-based cross-linking agents can not only fabricate stable cross-linked networks, but also interact with template proteins via multiple non-covalent interactions, thereby improving the recognition performance of imprinted sites [51].

**Figure 7:**
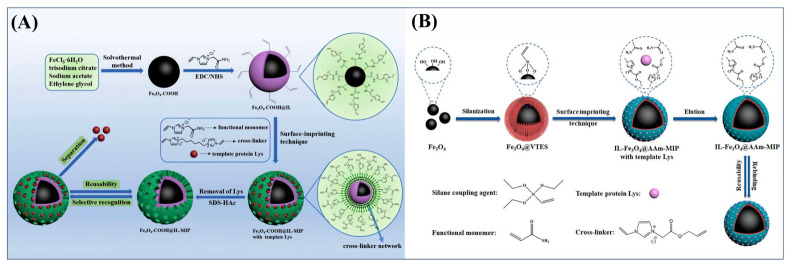
(**A**) Schematic diagram of the synthesis of Fe_3_O_4_-COOH@IL-MIP. (**B**) Schematic diagram of the synthesis of IL-Fe_3_O_4_@AAm-MIP. Reproduced with permission from [52,53], Elsevier, 2019/Elsevier, 2022.

As shown in Figure 7A, Wei et al. used the ionic liquid 1-vinyl-3-carbamoylmethylimidazolium chloride ([VAFMIM]Cl) as a functional monomer and the ionic liquid 1,6-hexanediyl-3,3′-bis-1-vinylimidazolium dichloride ([C_6_(VIM)_2_]Cl_2_) as a cross-linker. Using lysozyme (Lys) as the template protein, they prepared magnetic core–shell molecularly imprinted microspheres (Fe_3_O_4_-COOH@IL-MIP) via surface imprinting technology [52]. The Fe_3_O_4_-COOH@IL-MIP exhibited a maximum adsorption capacity of 166.36 mg/g for Lys and an imprinting factor of 2.67 [52]. Xu et al. used Fe_3_O_4_ nanoparticles as the support, acrylamide as the functional monomer, the ionic liquid 1-(α-allylacetate)-3-vinylimidazolium chloride ([AVIM]Cl) as the cross-linker, and lysozyme as the template to prepare magnetically responsive ionic liquid-cross-linked molecularly imprinted polymers (IL-Fe_3_O_4_@AAm-MIP), as shown in Figure 7B [53]. The results demonstrated that the prepared IL-Fe_3_O_4_@AAm-MIP achieved a high adsorption capacity of 334.1 mg/g and an imprinting factor of 2.94. This study confirmed that ionic liquids, when used as cross-linkers, provide multiple interaction sites, thereby significantly enhancing the adsorption capacity and selectivity of imprinted materials toward target proteins [53].

Therefore, ionic liquid-based cross-linking agents can construct homogeneous and stable polymer networks and maintain the structural stability of imprinted cavities. Meanwhile, they synergistically strengthen interactions with template proteins, compensate for the deficiencies of conventional cross-linking agents, and remarkably improve the adsorption capacity and selectivity of imprinted materials.

### 3.4. Ionic Liquids as Emulsifiers

**Figure 8:**
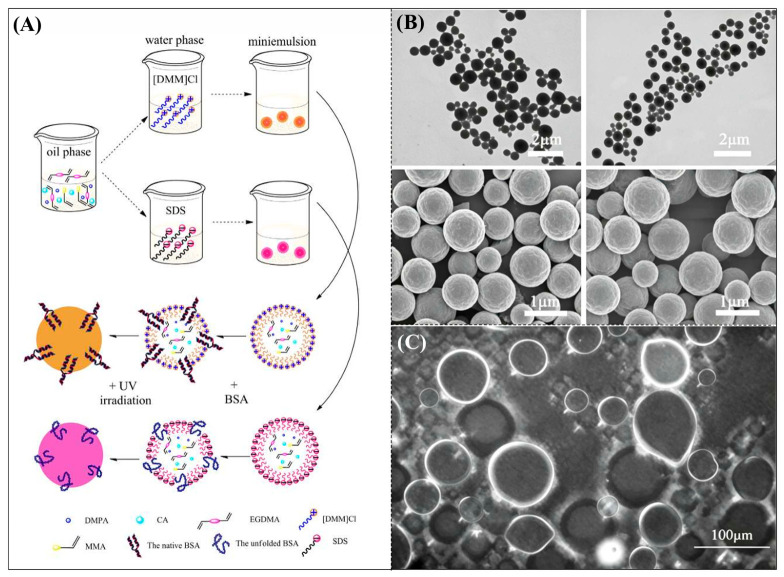
(**A**) Preparation process of interfacial molecularly imprinted microspheres. (**B**) SEM and TEM images of INIMs-[DMM]Cl. (**C**) Fluorescence spectrum of the emulsion stabilized by the ionic liquid surfactant, where green represents the distribution of FITC-labeled BSA protein. Reproduced with permission from [55], Elsevier, 2017.

Traditional emulsifiers such as sodium dodecyl sulfate (SDS) tend to disrupt protein conformation and impair the structural integrity of template proteins [54]. Amphiphilic ionic liquids possess both surface activity and biocompatibility, which can not only stabilize the emulsion system but also protect the native conformation of proteins [26,28]. Hu’s group designed and synthesized the amphiphilic ionic liquid 1-hexadecyl-3-vinylimidazolium bromide ([C_16_VIM]Br) as an emulsifier, which simultaneously played the dual roles of stabilizing the emulsion and providing recognition sites, successfully leading to the preparation of lysozyme molecularly imprinted microspheres, as shown in Figure 8 [55]. As a key emulsifier in the system, [C_16_VIM]Br, by virtue of its amphiphilic structure, tightly anchors the oil-water interface through its hydrophobic long alkyl chain, effectively reducing interfacial tension and stabilizing the water-in-oil miniemulsion system, thereby preventing emulsion stratification and coalescence. This provides a uniform and stable reaction environment for the imprinting polymerization, fundamentally solving the problems of conventional emulsifiers such as residual contamination and disruption of protein conformation. At the same time, the imidazolium cation structure of this ionic liquid can form multiple interactions with lysozyme, including electrostatic forces, hydrogen bonding, and π-π stacking, also serving as a functional monomer. The resulting imprinted microspheres exhibited uniform particle size (150–200 nm), good dispersity, a maximum adsorption capacity of 128.5 mg/g for lysozyme, and an imprinting factor of 2.73. Moreover, after eight adsorption–desorption cycles, the adsorption performance remained above 90% [55]. Therefore, when serving as emulsifiers, ionic liquids can stabilize the polymerization system and avoid protein denaturation induced by conventional emulsifiers. Meanwhile, they further improve the imprinting performance through multi-site interactions [42].

### 3.5. Ionic Liquids as Structure Stabilizers for Template Proteins

**Figure 9:**
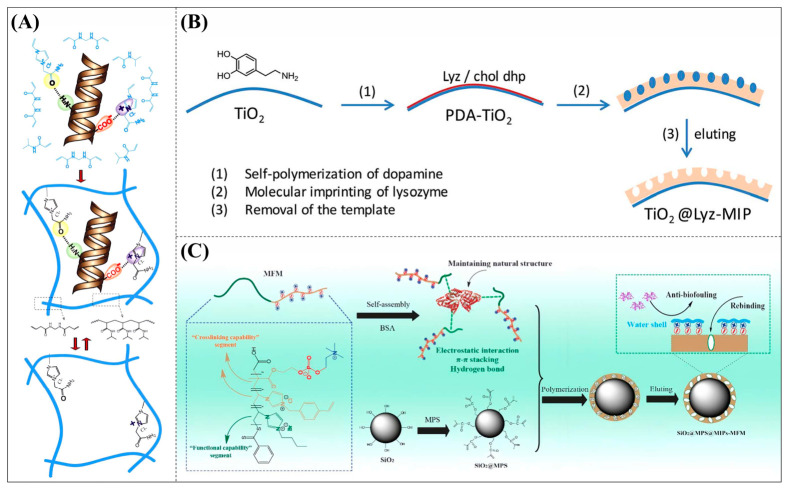
(**A**) Schematic diagram of the synthesis of MIH. (**B**) Preparation of lysozyme-imprinted TiO_2_ nanoparticles (TiO_2_@Lyz-MIPs). (**C**) Schematic diagram of the synthesis of SiO_2_@MPS@MIPs-MFM. Reproduced with permission from [56,57,58], Elsevier, 2014; Royal Society of Chemistry, 2019; American Chemical Society, 2021.

In accordance with the Hofmeister series, ionic liquids containing chaotropic cations and kosmotropic anions can effectively stabilize biomacromolecules. The introduction of such ionic liquids into protein imprinting systems can maintain the native conformation of proteins and improve imprinting accuracy [25,28]. Qian et al. designed an ionic liquid with both biocompatibility and polymerizability-1-vinyl-3-carbamoylmethylimidazolium chloride ([VAFMIM]Cl). It was found that the kosmotropic chloride ions in this ionic liquid effectively compete with water molecules, reducing the solvent-exposed surface area of the protein, thereby significantly stabilizing the α-helical structure of BSA, as shown in Figure 9A [56]. Circular dichroism spectroscopy confirmed that the introduction of [VAFMIM]Cl enabled the protein to maintain higher structural integrity in the pre-polymerization system, and the resulting imprinted hydrogel exhibited excellent recognition ability toward the target protein, with an imprinting factor of 2.66 [56]. On this basis, Zhao et al. further used choline dihydrogen phosphate (Chol DHP) as a conformation stabilizer for the template protein lysozyme (Lys) to construct imprinted polymers on the surface of dopamine-modified titanium dioxide nanoparticles, as shown in Figure 9B [57]. Circular dichroism results indicated that this ionic liquid effectively preserved the native conformation of Lys during polymerization. The imprinted nanoparticles achieved an imprinting factor as high as 4.40 for Lys and successfully separated the target protein from diluted egg white, demonstrating the application potential of ionic liquid-assisted surface imprinting in complex biological samples [57]. Meanwhile, Song et al. combined the concept of ionic liquid-mediated protein stabilization with green solvent design to construct ionic liquid-based magnetic molecularly imprinted polymers (SiO_2_@MPS@MIPs-MFM), as illustrated in Figure 9C [58]. This system fully exploited the stabilizing effect of ionic liquids on proteins and their multiple interactions with the template protein, generating a large number of high-affinity imprinted cavities. The resulting material exhibited a saturation adsorption capacity of 229.5 mg/g for bovine hemoglobin and an exceptionally high imprinting factor of 21.89, and successfully enabled selective enrichment of the target protein from bovine blood samples [58]. Consequently, ionic liquids maintain the native conformation of proteins via the synergistic interaction between chaotropic cations and kosmotropic anions, and prevent imprinted site mismatch induced by template denaturation, acting as a crucial additive for enhancing the efficiency of protein imprinting [25,28].

### 3.6. Ionic Liquids as Porogens

Pore-forming agents directly determine the pore structure, specific surface area, mass transfer property and accessibility of recognition sites of imprinted materials [59,60]. Conventional organic solvent porogens are confronted with inevitable drawbacks, including solvent residue, poor environmental compatibility and disruption of protein structure [61]. As green pore-forming agents, ionic liquids can precisely regulate the pore structure of imprinted materials and solve the core challenge of high mass transfer resistance of macromolecular proteins [28]. In a review, Ding et al. systematically concluded that ionic liquids, acting as porogens, can significantly enhance the pore structure, morphology, and mass transfer performance of imprinted materials by modulating the phase separation behavior during polymerization, providing an important theoretical basis for optimizing the pore structure of protein-imprinted materials [28,62].

## 4. Interaction Mechanism of Ionic Liquid-Based Protein-Imprinted Materials

### 4.1. Ionic Liquid–Template Protein Interactions

The ability of ionic liquids to play multifunctional roles and significantly enhance the performance of imprinted materials in protein imprinting systems originates fundamentally from their precisely tunable, multiple non-covalent interactions with proteins; in some systems, reversible covalent interactions can also be incorporated to form a combined recognition mode [26,28]. The high designability of the cationic and anionic structures of ionic liquids enables the formation of stable intermolecular forces with amino acid residues and functional groups on the protein surface, which mainly comprise four core types: electrostatic interactions, hydrogen bonding and hydrophilic interactions, hydrophobic interactions, and π-π stacking interactions [28]. The combination and strength of these different forces directly determine template immobilization efficiency, imprinting site precision, and protein conformational stability.

Electrostatic interactions arise from the attraction between charged cations or anions of ionic liquids and charged amino acid residues on the protein surface, providing the driving force for initial template recognition and positioning. Hydrogen bonding and hydrophilic interactions are characterized by the formation of specific hydrogen bonds between polar groups of ionic liquids (e.g., C_2_-H of imidazolium rings, amide groups, hydroxyl groups) and amide bonds in the protein backbone or polar side-chain residues, as well as extensive hydrophilic association on the protein surface via dipole–dipole, ion-dipole and other polar interactions. As a fundamental and dominant force in the interactive system, hydrophilic interactions act synergistically with hydrogen bonding, which not only fundamentally ensures the aqueous compatibility of the imprinting system and stabilizes the native conformation of template proteins, but also significantly strengthens the stability of pre-polymerization complexes and greatly improves the recognition specificity of imprinted sites for target proteins. The contribution of hydrophobic interactions is modulated by the alkyl chain length of the ionic liquid cation: with short chains, the effect is weak; as chain length increases, the nonpolar tail tends to associate with hydrophobic patches on the protein surface or internal hydrophobic regions, thus strengthening binding affinity. Furthermore, the π-system of aromatic heterocycles such as the imidazolium ring in ionic liquids can engage in π-π stacking with aromatic amino acid residues including phenylalanine, tyrosine, and tryptophan of the protein, further contributing to the binding free energy through face-to-face or edge-to-face configurations [26,28].

Furthermore, the various interactions between ionic liquids and template proteins do not act in isolation but often work synergistically [26,28]. The synergistic mechanism of non-covalent interactions plays a dominant role: electrostatic interactions provide initial anchoring and spatial orientation; hydrogen bonding and hydrophilic interactions guarantee recognition specificity and complex stability; hydrophobic interactions and π-π stacking interactions further enhance binding affinity and protein conformational stability. The combination, matching, and dynamic balance of these multiple forces collectively determine the template immobilization efficiency, imprinting site precision, and selective recognition performance of the materials [28,33]. To facilitate an intuitive understanding of the structure-interaction-performance relationship, Table 2 concisely summarizes the ionic liquids, their dominant non-covalent interaction types, and key performance indicators.

### 4.2. Research Methods for Ionic Liquid-Protein Interactions in Protein Imprinting

In-depth understanding of the interaction mechanism between ionic liquids and proteins is fundamental to the design of ionic liquid-assisted protein-imprinted materials. To date, researchers have primarily employed two approaches—theoretical calculations and experimental characterization—to reveal the nature and principles of these interactions from multiple perspectives, including energy, structure, dynamics, and thermodynamics [26,28].

#### 4.2.1. Experimental Methods

Systematic experimental investigation of the interactions between ionic liquids and proteins is essential for elucidating the mechanism of action of ionic liquids in protein imprinting systems, optimizing material design, and promoting practical applications. Experiments can directly characterize the existence and strength differences in non-covalent interactions—such as electrostatic forces, hydrogen bonding, hydrophobic effects, and π-π stacking—providing direct evidence for the stabilization of protein conformation and protein anchoring by ionic liquids [26]. Using methods such as spectroscopy, electrochemistry, and thermodynamics, it is also possible to quantitatively assess the influence of ionic liquid structures on the secondary and tertiary conformations of proteins as well as on their active sites, thereby elucidating the inherent principles underlying protein protection and denaturation inhibition [8,33], and avoiding biases that may arise from purely theoretical predictions. Meanwhile, experiments can screen ionic liquid structures suitable for specific target proteins and determine optimal interaction conditions, providing a reliable basis for the rational design of high-performance protein-imprinted materials. An in-depth understanding of the above interaction principles can help address the bottlenecks of traditional imprinting, such as conformational instability, poor specificity, and slow mass transfer, and is of significant theoretical and practical value for improving protein recognition accuracy and expanding the practical applications of ionic liquid-based imprinted materials in fields such as biosensing and separation/purification [26,28]. Representative experimental methods include the following. Fluorescence spectroscopy exploits the sensitivity of endogenous tryptophan or tyrosine residues to the microenvironment to quantitatively analyze binding constants, number of binding sites, and quenching mechanisms through changes in fluorescence intensity or emission wavelength. Isothermal titration calorimetry directly measures thermodynamic parameters (binding constant, stoichiometry, enthalpy change, and entropy change) during the binding process to determine the driving force type of the interaction. Circular dichroism spectroscopy monitors far-UV CD spectra to quantitatively analyze changes in secondary structure content (e.g., α-helix, β-sheet) and to evaluate the effect of ionic liquids on protein conformational stability [57]. Nuclear magnetic resonance spectroscopy provides atomic-level resolution information; saturation transfer difference NMR can identify protons in ionic liquid molecules that participate in interactions and map the binding epitope. Surface plasmon resonance enables real-time monitoring of binding kinetics, obtaining association rate constants, dissociation rate constants, and equilibrium dissociation constants, thereby quantitatively evaluating the influence of different ionic liquid structures on binding affinity, and serving as an effective means for screening high-performance functional monomers. The above theoretical and experimental methods complement each other and are used synergistically to comprehensively reveal the molecular mechanisms of ionic liquid-protein interactions from the perspectives of energy, structure, dynamics, and thermodynamics, providing important theoretical guidance for the rational design of functional ionic liquids and the development of high-performance protein-imprinted materials [28,33].

#### 4.2.2. Theoretical Calculations

Currently, the theoretical calculation methods used to study the interactions between ionic liquids (ILs) and proteins mainly include molecular dynamics (MD) simulations, density functional theory (DFT), quantum chemical calculations, and molecular docking [26].

**Figure 10:**
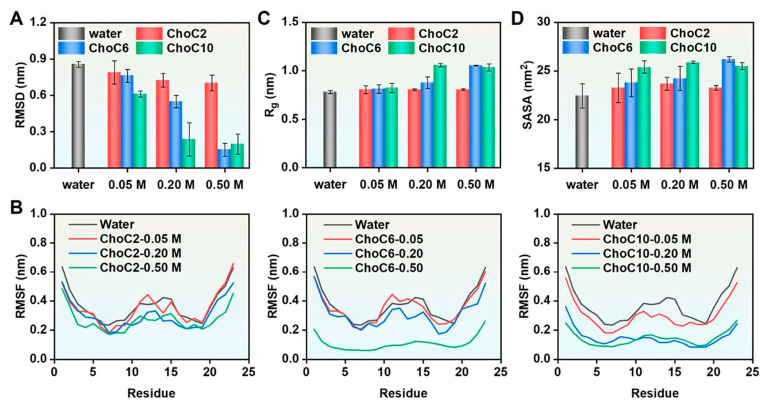
Molecular dynamics analysis of Mag2 in the ChoCn ionic liquid system: (**A**) average backbone RMSD; (**B**) residue-specific RMSF; (**C**) average radius of gyration; (**D**) SASA values. Reproduced with permission from [67]. Elsevier, 2025.

Among these methods, molecular dynamics simulations can dynamically model the interaction process between ILs and proteins under different conditions, accurately capturing conformational changes, evolution of binding sites, and movement trajectories, thereby clarifying the regulatory effects of ILs on the secondary and tertiary structures of proteins. For example, Gao et al. used molecular dynamics simulations to reveal that the anion of cholinium hexanoate (ChoC_6_) forms a stable hydrogen bonding network with the peptide backbone of the antimicrobial peptide Magainin 2 through its carboxyl group, thereby effectively maintaining the α-helical conformation and reducing the competitive interference of water molecules with the internal hydrogen bonds of the protein [67], as shown in Figure 10.

**Figure 11:**
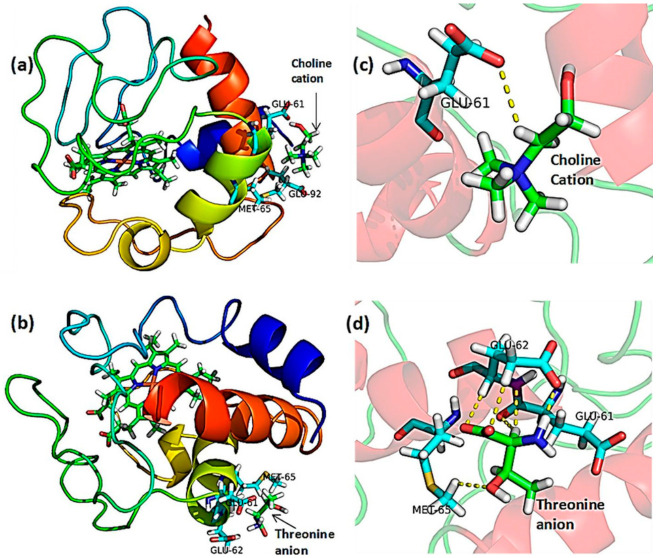
Cartoon view for the most stable (energetically) docking positions of segregate interactions of Cyt c protein with [Ch][Thr] IL ions: (**a**) Choline cation interacting at Cyt c surface, (**b**) Expanded view of choline cation showing hydrogen bonds with Glu61 residue of Cyt c, (**c**) Threonine anion interacting at Cyt c surface, (**d**) Expanded view of threonine anion showing hydrogen bonds with Glu61, Glu62 and Met65 residues of Cyt c. Reproduced with permission from [69], Wiley-VCH, 2020.

Density functional theory and quantum chemical calculations can quantitatively compute the interaction energies of non-covalent interactions between ILs and proteins, including electrostatic forces, hydrogen bonding, hydrophobic effects, and π-π stacking, thereby clarifying the contribution of each type of interaction and revealing the intrinsic nature by which ILs stabilize protein conformation and achieve protein anchoring. Dong et al. systematically studied six amino acid-based imidazolium ionic liquids using ab initio molecular dynamics. Radial distribution function and combined distribution function analyses revealed that cation-anion interactions are dominated by π-π stacking, with additional hydrogen bonding networks—most notably between the carboxylic acid group of the anion and the hydrogen atoms of the imidazolium ring. This study directly demonstrates that the synergy between π-π stacking and hydrogen bonding is key to the structural stability of ionic liquid systems [68]. Molecular docking, on the other hand, enables rapid screening of the optimal binding sites between ILs and proteins and predicts their binding modes, offering efficient guidance for the structural design of ILs tailored to specific proteins. Sahoo et al. employed molecular docking to study the interaction of a threonine-based ionic liquid with cytochrome c. They found that both the cation and anion of the ionic liquid bind in the cavity between the surface helix and loop of the protein, with binding free energies ranging from −9 to −18 kJ/mol. Moreover, the cation forms hydrophobic contacts with residues through its alkyl chain, whereas the anion relies solely on hydrogen bonding [69], as shown in Figure 11.

As an important complement and extension of experimental research, theoretical calculation methods play an irreplaceable role in the study of interactions between ILs and proteins, providing accurate theoretical support for in-depth elucidation of the nature of these interactions and for the optimization of system design. Compared with experimental approaches, theoretical calculations can overcome the limitations of experimental conditions, directly revealing the microscopic mechanisms of IL-protein interactions at the molecular and atomic levels, thereby effectively compensating for the shortcomings of experimental methods in capturing short-range interactions, dynamic processes, and quantitative analysis of interaction energies [26,28].

## 5. Applications of Ionic Liquid-Assisted Protein-Imprinted Materials

### 5.1. Bioseparation and Purification

Ionic liquid-based protein-imprinted materials achieve highly selective capture of target proteins through multiple non-covalent interactions, making them particularly suitable for the enrichment of low-abundance proteins in complex biological samples [28,33]. Liu et al. used a deep eutectic solvent as both a functional monomer and a cross-linker to construct magnetic molecularly imprinted polymers, successfully achieving highly selective purification of BHb from calf serum samples [47].

In biological separation and purification, ionic liquid-based imprinted materials not only achieve efficient template immobilization through multiple non-covalent interactions, thereby enhancing imprinting site density and recognition accuracy, but also enable the modulation of material pore structures by ILs, improving mass transfer efficiency for macromolecular proteins. Furthermore, the combination of ionic liquids with magnetic nanoparticles facilitates rapid separation and reusability, providing a feasible technical route for the efficient and highly selective isolation of target proteins from complex biological samples [28,33].

### 5.2. Biosensing and Detection

The excellent conductivity, film-forming ability, and biocompatibility of ionic liquids make them ideal materials for constructing high-performance protein sensors [28]. Aubé et al. self-assembled an alkylimidazolium-based ionic liquid into a monolayer on a gold surface to construct an SPR sensing platform for the detection of the breast cancer marker HER2, obtaining binding kinetic parameters in cell lysates and finding that the ionic liquid surface significantly reduced nonspecific adsorption [70]. Furthermore, fluorescent sensors constructed by combining ionic liquid-based imprinted materials with quantum dots achieve detection limits at the picomolar level [57]. In the field of electrochemical biosensing, ionic liquid-based molecularly imprinted polymers have demonstrated particularly outstanding performance. For example, Wong and colleagues developed a carbon paste electrode modified with ionic liquids and magnetic molecularly imprinted polymers for the selective detection of sarcosine, a prostate cancer biomarker, achieving a detection limit of 5.1 × 10^−8^ mol·L^−1^ in biological samples with recoveries ranging from 96% to 104% [71]. Ghorbanizamani et al. constructed an electrochemical sensor for the hepatocellular carcinoma biomarker alpha-fetoprotein using a screen-printed gold electrode modified with hydroxyapatite, nano-TiO_2_, and the ionic liquid 1-butyl-3-methylimidazolium bis(trifluoromethanesulfonyl)imide. The sensor exhibited a wide linear range of 0.01–400 ng·mL^−1^ and an ultralow detection limit of 0.058 ng·mL^−1^ in saliva samples [72].

In biosensing, ionic liquid-based imprinted materials not only enhance the binding stability between the imprinted material and the electrode interface, thereby improving sensor reusability, but also the conductivity of ionic liquids amplifies the electrochemical signal, increasing detection sensitivity. Moreover, the multiple interactions of ionic liquids can endow sensors with environmentally responsive properties, enabling smart sensing [28,33].

### 5.3. Disease Diagnosis

Ionic liquid-based imprinted materials can serve as “artificial antibodies” for recognizing disease-related protein biomarkers, showing promise for the early diagnosis of cancer, cardiovascular diseases, and infectious diseases [28,33]. For example, imprinted materials based on the (Cys)VIMBF_4_ ionic liquid have been used for the recognition of alpha-fetoprotein [73]; those based on CCPEimBr for the detection of human epididymis protein 4 [74]; and those based on PMIMBr for the recognition of progastrin-releasing peptide [75]. In the field of infectious disease diagnosis, imprinted materials based on the [Bvim]Br ionic liquid can specifically recognize the SARS-CoV-2 spike protein, enabling rapid virus detection at a lower cost than traditional antibodies [76]. For cardiovascular disease diagnosis, ionic liquid-based imprinted materials have been applied to the detection of cardiac troponin, where the design of the ionic liquid structure can enhance the capture ability for low-abundance biomarkers [28].

In disease diagnosis, ionic liquid-based imprinted materials can not only replace traditional antibodies, overcoming the drawbacks of high cost, poor stability, and batch-to-batch variation associated with antibodies, but also their multiple interactions enhance the capture ability for low-abundance biomarkers. Furthermore, ionic liquids are compatible with various detection platforms, enabling high-sensitivity detection [28,33].

### 5.4. Disease Therapy

Ionic liquid-based imprinted materials can serve as targeted drug delivery vehicles or signal-modulating elements, enabling precision therapy of diseases [8,28]. An epitope-imprinted polymer targeting HER2 achieved targeted drug delivery in a mouse model of ovarian cancer, significantly inhibiting tumor growth [8]. The introduction of ionic liquids further enhances the binding strength of imprinted materials to target cells, improving delivery efficiency [28]. In antiviral therapy, glyco-imprinted nanoparticles specifically recognize viral envelope glycoproteins, blocking virus–host cell binding and inhibiting HIV-1 infection [28]. When combined with photothermal materials, ionic liquid-based imprinted materials can selectively bind to cancer cell surface markers and generate localized hyperthermia upon near-infrared irradiation, achieving precise photothermal ablation while minimizing damage to surrounding normal tissues [28].

In disease therapy, ionic liquid-based imprinted materials can achieve highly specific recognition of diseased cells through structural design, thereby reducing damage to normal cells. At the same time, the multiple interactions of ionic liquids enhance drug loading and controlled release capabilities. Furthermore, ILs can synergize with various therapeutic modalities—such as chemotherapy, photothermal therapy, and immunotherapy—enabling combination therapy [8,28].

In summary, ionic liquid-based protein-imprinted materials, leveraging their core advantages—such as high structural designability, synergistic multiple non-covalent interactions, good biocompatibility, and excellent conformational stabilization ability—exhibit broad application prospects in four major fields: bioseparation and purification, biosensing and detection, disease diagnosis, and disease therapy. The multifunctional roles of ionic liquids in protein imprinting technology have laid a solid foundation for advancing this technology from laboratory research to practical applications in clinical diagnosis and biomedicine.

## 6. Conclusions and Prospects

Owing to their prominent structural designability, abundant multiple interaction sites, and excellent biocompatibility, ionic liquids provide a novel technical route for constructing high-performance protein imprinted materials by stabilizing the native conformations of proteins, reinforcing specific interactions, and optimizing the microenvironment of imprinting interfaces [26,28]. Meanwhile, this technique delivers a significant contribution to green chemistry by utilizing low-toxicity and biodegradable ionic liquids and deep eutectic solvents as eco-friendly alternatives to conventional volatile organic solvents, thereby enabling sustainable and environmentally benign protein separation and enrichment. The integration of functionalized ionic liquids with nanoporous supports, surface imprinting, and template immobilization strategies can significantly enhance the adsorption capacity, selectivity, and reusability of imprinted materials toward target proteins, demonstrating great potential in the separation of complex biological samples [33,41,47]. Through in-depth summary and analysis of the molecular design, preparation optimization, and interaction mechanisms of ionic liquids used as functional monomers, cross-linkers, emulsifiers, structure stabilizers, and porogens, this review reveals that ionic liquid-based protein imprinting technology, as a highly efficient and green strategy for biomolecule separation and enrichment, has successfully overcome the inherent bottlenecks of conventional protein imprinted materials in terms of specificity, mass transfer efficiency, and protein conformational stability [28,33].

Collectively, previous review studies have established a fundamental theoretical framework and outlined the general research progress of ionic liquid-mediated protein imprinting. Nevertheless, centering on the essential requirement of rational material design for such systems, this review constructs a more integrated and systematic research paradigm. It elaborates the multifaceted and comprehensive roles of ionic liquids throughout the entire protein imprinting workflow, deeply clarifies the synergistic regulatory mechanism of multiple non-covalent interactions on imprinting site fidelity and the natural conformational stability of template proteins, and comprehensively summarizes the state-of-the-art advances of this technology spanning the full research chain from bioseparation to clinical diagnosis and therapy over the period of 2020–2026. This work effectively compensates for the insufficient systematic elaboration and in-depth mechanistic discussion in existing reviews. The established structure-activity relationships and mechanistic insights in this review provide robust theoretical guidance for the rational design and practical implementation of high-performance protein imprinting materials.

Nevertheless, translating this technology from fundamental research into clinical and industrial practice requires both a focus on high-impact application scenarios and a systematic resolution of the persistent challenges that remain. Among the most promising scenarios, the enrichment of low-abundance protein biomarkers—such as cytokines, phosphoproteins, and circulating tumor cell markers—from complex biofluids stands out as a particularly powerful direction. In this area, ionic liquid-based protein-imprinted polymers are expected to outperform conventional antibodies owing to their lower cost, superior stability, and excellent reusability [28,32]. Secondly, membrane proteins are notoriously difficult to isolate because of their hydrophobicity and conformational flexibility; ionic liquids, with their ability to stabilise amphipathic structures and to provide multiple non-covalent anchoring points, are poised to offer a unique advantage in this domain [8,11]. Thirdly, although in vivo sensing is still at an early stage, the development of biocompatible and stimuli-responsive ionic liquid components is making real-time monitoring of protein biomarkers in interstitial fluid or blood increasingly feasible—provided that the issues of long-term stability and immune compatibility are adequately resolved [77].

Concurrently, several major challenges must be overcome to enable practical translation. The first is scalable preparation. Current production of protein-imprinted polymers is largely confined to milligram-to-gram scales in batch reactions. To meet industrial demands for large-volume protein separation, it is urgent to develop reproducible, continuous, and cost-effective manufacturing processes—for example, flow-based imprinting and spray drying [7,11]. The second challenge concerns ionic liquid toxicity. Although many functional ionic liquids have been designed with biocompatible cations such as cholinium and anions such as chloride or dihydrogen phosphate, systematic data on chronic exposure, biodegradability, and ecotoxicological profiles remain conspicuously scarce [78]. Rational selection or de novo design of intrinsically low-toxicity ionic liquids is a mandatory prerequisite before any therapeutic or injectable application. The third challenge is bio-fouling and nonspecific adsorption. In complex biological matrices such as serum, plasma, and cell lysates, nonspecific protein adsorption severely compromises both selectivity and reusability. Future materials should integrate anti-fouling moieties—such as zwitterionic groups or poly(ethylene glycol) chains—into the ionic liquid backbone or the imprinting layer, and systematic benchmarking against state-of-the-art low-fouling surfaces is urgently needed [79].

Overcoming these challenges will not only accelerate the translation of ionic liquid-mediated protein imprinting technologies but also unlock their full potential in precision diagnostics, targeted therapy, and biomanufacturing. In summary, with the continuous deepening of fundamental research and the integration of interdisciplinary technologies, ionic liquid-based protein imprinting technology will undoubtedly play an increasingly vital role in bioanalysis, clinical diagnosis, drug development, food safety and other fields, providing more efficient and intelligent solutions for proteomics research and the development of related industries [28,33].

## Figures and Tables

**Table 1:** Multifunctional roles, functions and key advantages of ionic liquids in protein imprinting systems.

Functional Role	Core Function	Key Advantages
Protein Anchoring Site	Mediates multiple non-covalent interactions and directionally immobilizes template proteins	Improves protein loading capacity and site uniformity, and reduces non-specific adsorption
Functional Monomer	Constructs specific recognition sites and strengthens target protein affinity interactions	Broadens interaction mechanisms, enhances aqueous compatibility, and improves recognition selectivity
Cross-Linker	Builds a stable polymer network and preserves the structure of imprinted cavities	Optimizes cross-linking homogeneity and reinforces the structural stability of the material
Emulsifier	Stabilizes the emulsion polymerization system and tailors the morphology and particle size of microspheres	Prevents protein conformational denaturation and improves the controllability of the polymerization system
Protein Conformation Stabilizer	Maintains the native conformation of template proteins and suppresses denaturation and inactivation	Guarantees precise matching of imprinted sites and improves imprinting fidelity
Pore-Forming Agent	Regulates the pore structure of the material and facilitates macromolecular mass transfer	Increases specific surface area and site accessibility, and accelerates adsorption kinetics

**Table 2:** Summary of Ionic Liquid Monomers, Primary Interaction Types, and Key Performance Indicators in Protein Imprinting Systems.

IL Monomers	Structures of Monomers	Template Protein	Primary Interaction Types	Q_e_ (mg/g)	IF	Selectivity	Ref.
[TMSPMIM]Cl	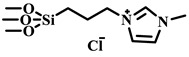	BSA	H-bond & hydrophilic Electrostatic π-π stacking	50.6	3.33	β = 4.97 (vs. Lyz), α =2.53 (vs. OVA)	[41]
[VAFMIM]Cl	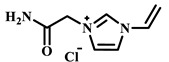	BSA	H-bond & hydrophilic Electrostatic Hydrophobic π-π stacking	741.5	1.65	β = 1.39 (vs. Lyz), β = 1.30 (vs. BHb), β = 1.35 (vs. Cyt c)	[35]
[P_4,4,4,4_][SS]	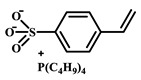						
[VAFMIM]Cl	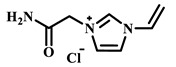	BSA	H-bond & hydrophilic Electrostatic π-π stacking	150.2	5.17	α = 11.83 (vs. BHb), α = 9.39 (vs. Lys), β = 23.97 (BSA/BHb mixture)	[48]
VACM	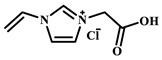	BSA	H-bond & hydrophilic Electrostatic Hydrophobic π-π stacking	24.30	1.31	β = 1.10 (vs. Lyz), β = 1.58 (vs. OVA)	[63]
VBCM	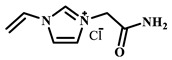			18.1	1.27	β = 1.43 (vs. Lyz), β = 1.10 (vs. OVA)	
VSPM	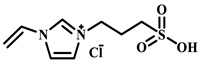			9.3	4.52	β = 4.30 (vs. Lyz), β = 6.19 (vs. OVA),	
[VDAIM]Cl	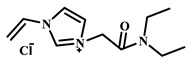	BSA	H-bond & hydrophilic Electrostatic Hydrophobic π-π stacking	502.5 ± 27	6.27	α = 5.57 (BSA/BHb mixture), β = 6.37 (permeation selectivity)	[46]
[AMIM][PF_6_]	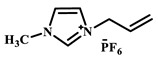	BSA	H-bond & hydrophilic Electrostatic Hydrophobic π-π stacking	116.4	2.96	β = 10.10 (BSA/BHb mixture)	[64]
[VAFMIM]Cl	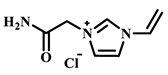	Lys	H-bond & hydrophilic Electrostatic Hydrophobic π-π stacking	166.4	2.67	R = 1.92 (vs. OVA)	[52]
[HBVMIM]Cl_2_	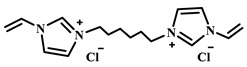						
[AVIM]Cl	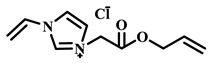	Lys	H-bond & hydrophilic Electrostatic Hydrophobic π-π stacking	334.1	2.94	β = 1.69 (vs. BHB), β = 15.47 (vs. BSA), β = 1.18 (vs. Cyt c), β = 3.38 (vs. OVA)	[53]
[AAPIM]Cl	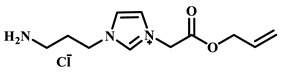	Lys	H-bond & hydrophilic interaction Electrostatic π-π stacking	213.7	2.02	R = 2.97 (vs. BSA), R = 3.72 (vs. OVA)	[45]
[Ch][dhp]	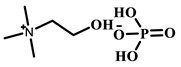	Lyz	Physical H-bond & hydrophilic Electrostatic	43	2.36	β = 2.21 (vs. Cyt c), α = 4.30 (Lys/Cyt c mixture)	[65]
SiO_2_-MPS/IL	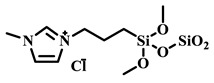	Hb	Coordination H-bond & hydrophilic Electrostatic	67.7	5.0	Significantly higher selectivity vs. BSA/Cyt c/Lyz	[66]
PBA	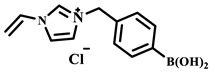	OVA	H-bond & hydrophilic π-π stacking Covalent (boronate ester)	571	5.09	β = 7.68 (vs. HRP)	[40]

## Data Availability

No new data were created or analyzed in this study. All data cited and discussed in the manuscript are available from the published literature.
